# Evaluating Elastic-Plastic Wavy and Spherical Asperity-Based Statistical and Multi-Scale Rough Surface Contact Models with Deterministic Results

**DOI:** 10.3390/ma14143864

**Published:** 2021-07-10

**Authors:** Nolan Ryan Chu, Robert L. Jackson, Xianzhang Wang, Arup Gangopadhyay, Hamed Ghaednia

**Affiliations:** 1 Mechanical Engineering Department, Auburn University, Auburn, AL 36849, USA; nrc0011@auburn.edu; 2 School of Mechanical and Power Engineeing, Guangdong Ocean University, Zhanjiang 524088, China; wangxianzhang@gdou.edu.cn; 3 Ford Motor Company, Dearborn, MI 48124, USA; agangopa@ford.com; 4 Gehring L. P., Farmington Hills, MI 48335, USA; hamed.ghaednia@gehring-group.com

**Keywords:** roughness, mechanics, joint stiffness

## Abstract

The solution to an elastic-plastic rough surface contact problem can be applied to phenomena such as friction and contact resistance. Many different types of models have therefore been developed to solve rough surface contact. A deterministic approach may accurately describe the entire surface, but the computing time is too long for practical use. Thus, mathematically abbreviated models have been developed to describe rough surface contact. Many popular models employ a statistical methodology to solve the contact problem, and they borrow the solution for spherical or parabolic contact to represent individual asperities. However, it is believed that a sinusoidal geometry may be a more realistic asperity representation. This has been applied to a newer version of the stacked multiscale model and statistical models. While no single model can accurately describe every contact problem better than any other, this work aims to help establish guidelines that determine the best model to solve a rough surface contact problem by applying mathematical and deterministic models to two reference surfaces in contact with a rigid flat. The discrepancies and similarities form the basis of those guidelines.

## 1. Introduction

Contact between rough surfaces is a ubiquitous problem that can be applied to numerous phenomena such as friction, wear, and contact resistance. It can be modeled in many ways such as statistical [1,2,3,4], fractal [5], and multi-scale [6] models. In the statistical model, the surface is generalized by using mathematical parameters to calculate probabilities to determine the contact area and force. Fractal-based models account for different scales of surface features neglected by statistical models. Due to their limitations, such as predicting zero contact area, for a true fractal surface, they are not considered in this work. The multi-scale model more accurately incorporates deformation mechanics and is not restrained to zero area of contact at the smallest scales, which occurs if perfect fractal surfaces are assumed.

Henrich Hertz was one of the first researchers in the field of contact mechanics. He solved the elastic deformation of a parabola in contact with a flat surface, which can be applied to cylindrical or spherical contact [7]. However, he did not consider the effects of friction or plastic deformation. By incorporating roughness models, his solution has been expanded from a single asperity, or raised point on a surface, to a system of asperities that describes a surface’s topography (roughness).

One such expansion is the statistical model provided by Greenwood and Williamson [1], which is referred to as the GW model. Their work considered the interaction between two planes. One plane was perfectly flat and rigid, while the other was covered with spherical asperities of different heights. They assumed that asperities behave independently of each other and deformation was restricted to the asperities. This model relies on the interference, or the material that deforms to maintain a given separation between the surfaces.

The GW model only considers elastic contact, so the model has been refined to include the effects of elastic-plastic deformation. One such model was derived by Jackson and Green [3] (JG), which establishes the required load above which the statistical model predicts plastic deformation. Other models such as those proposed by Chang, Etsion, and Bogy [2] (CEB) and Kogut and Etsion [4] (KE) that include the effects of plasticity are not considered here; however, we expect the current results using a spherical asperity model would be very similar to those predicted by the KE and related model. As the contact pressure increases, the internal stress within asperities also increases, which causes yielding and plastic deformation. At the critical interference, *ω_c_*, the material is assumed to yield. The JG model is limited to small deformations such that the contact radius is 41% or less of the radius of curvature. Wadwalker et al. [7] extend the model for larger contact radii, but asperities may behave like isolated spheres rather than peaks at higher loads.

Statistical models are reliable and easily implemented, but shortcomings exist. They assume a homogeneous radius of curvature for the entire region and neglect the effects of different scales of features. Many of them also neglect the coupling of deformation between asperities and the substrate. Ciavarella et al. developed a revised model that accounts for lateral asperity interaction. Afferrante et al. followed their approach with a coalescing asperity model, while Vakis expanded it below the mean asperity height [8,9,10]. This is similar to the use of a wavy surface model that includes lateral asperity interaction as explored in the current work. 

Majumdar and Bhushan [5] (MB) created a fractal model for rough surface contact. They applied the Weierstrauss–Mandelbrot (WM) function to multiple levels of roughness. While it depicts a different roughness for each scale, a surface may not have a spectrum that can be related to the fractal equation. Ciavarella et al. [11] solved a 2D W-M fractal-rigid flat interface using a stacked asperity assumption and an elastic sinusoidal model derived by Westergaard [12]. Morag and Etsion [13] did attempt to improve the model by allowing the asperity contacts to begin the elastic regime and become plastic as load is increased, but the fractal models are still arguably deficient in other ways. The usage of the fractal geometry for describing real rough surfaces is still the subject of debate and it is questionable to use them in a rough surface contact model [14,15,16,17,18,19,20]. In fact, it has been shown in elastic and elastic-plastic contact that a true fractal surface in contact will have zero contact area and infinite pressure [21,22]. There are also other families of models based on diffusion and fractal geometries, but it is unclear or impossible to employ different single asperity models within them. For these reasons, this work does not consider a fractal-based model.

To overcome the limitations of the GW model and predict a realistic area of contact, the multi-scale model as developed by Jackson and Streator [6] (JS) is used. Their model builds off Archard’s [23] “protuberance upon protuberance” concept in which the Hertzian sphere was expanded by including hemispheres of smaller radii on it. As loads increase, the surfaces come into complete contact at the smallest scales and begin compressing at larger scales. Archard’s theory predicted a linear relationship between area and force and that rougher surfaces would only flatten with larger force. Jackson and Streator refined Archard’s model so it could be applied to real surfaces [6]. They made the following assumptions: smaller asperities are stacked on larger asperities, load is distributed equally over all asperities on each scale, total load does not depend on scale, and the contact area is limited to that of the scale below. They applied the Johnson, Greenwood, and Higginson piecewise solution [23] for perfectly elastic 3D sinusoidal contact and connected the equations. To consider roughness, the surface was converted using a discrete Fourier transform into a series of sine waves of known frequency and amplitude [6].

The JS model was subsequently modified using results from Krithivasan and Jackson [24], who analyzed a finite element model of a sinusoidal asperity. Like the JG model, a critical value below which contact remains perfectly elastic exists. Because interference is not calculated, the critical values are found in terms of force.

Other recent works have also sought to incorporate the effect of coatings [25], size-dependent properties [26] (especially material strength), and tangential loading or friction [27,28,29] and even wear or surface damage [30]. However, it is difficult to consider these in a full deterministic rough surface contact model, and so the current work focuses on the normal loading of homogeneous rough surfaces.

Idealistically, deterministic models solve rough surface contact without making any significant simplifying assumptions (in contrast the mathematical and statistical models are already discussed). A review and summary of some deterministic rough surface contact modelling methods is provided in Liu et al. [31]. Later, Liu et al. [32] used the finite element method with plastic deformation and the simplex algorithm to consider cylindrical and 2-D rough surface contact in plane strain. Somewhat different from other works, several researchers [33,34,35] used a semi-analytical boundary element-based approach to solve the elastic-plastic problem. Finite elements were used by Pei et al. [36] and Sahoo and Ghosh [37] to consider the contact of self-affine fractal elastic-plastic surfaces. The rough surfaces of a microelectromechanical system in contact were considered by Liu et al. [38] using the finite elements. The finite element deterministic modeling methodologies were discussed by Thompson [39,40] in order to predict the thermal contact resistance. A deterministic finite element model was compared to a hybrid analytical model in the work by Megalingam and Mayuram [41]. More recently, Wang et al. [42] and An et al. [43] implemented an elastic-plastic finite element deterministic model of measured rough surfaces and sought to refine the mesh toward a converged solution. Although all of these models are referred to as deterministic, they still make many assumptions and contain errors in their predictions.

This paper accepts that no one model best describes every single contact mechanics problem; otherwise, developing differing theories would be pointless. However, it establishes parameter-based guidelines that will determine which model most accurately describes a given problem based on the predicted load and contact area for a given surface separation. In particular, it compares elastic-plastic sinusoidal and spherical shaped asperity models to determine which geometry is more effective when compared to a deterministic model.

## 2. Methodology

### 2.1. Applying the JG Asperity Model to the GW Model

The total contact area and load under the GW model are given as follows:

(1) $$A_{r}\left(h\right)=A_{n}\eta \int_{h}^{\infty }\bar{A}\left(z-h\right)\phi \left(z\right)dz,$$

(2) $$P\left(h\right)=A_{n}\eta \int_{h}^{\infty }\bar{P}\left(z-h\right)\phi \left(z\right)dz.$$

where *A_n_* is the nominal or apparent area of contact (before roughness is considered), *A_r_* is the real area of contact, *P* is the total contact force, *h* is the mean surface separation, *η* is the areal mean density, and *ϕ* is the asperity height distribution. Note that the macron above A and P denotes a prediction for a single asperity.

The asperities are assumed to be evenly distributed, homogenous, and with an RMS (root mean square) height *σ_s_*. To determine their areal density and height, they are manually counted by scanning the surface and identifying points where the height was higher than any neighboring point orthogonally or diagonally. The asperity radius is measured in two directions and averaged. The original work assumes elastic Hertz contact and that the asperities have a constant radius of curvature, but the equations in Appendix A are used because yielding occurs in most metallic contacts. The single asperity contact area and load are inserted into Equations (1) and (2) as functions of surface separation, and the integrals are evaluated to determine the total contact area and load between surfaces. These predictions will then be compared to the additional models described in the following sections.

### 2.2. Sinusoidal Asperities in the GW Model

**Figure 1:**
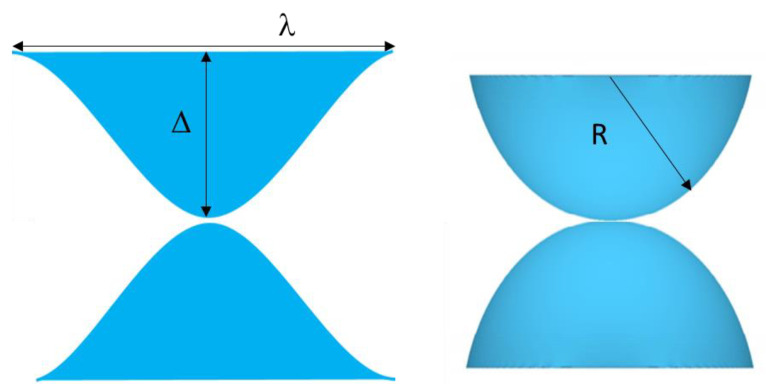
Geometry of sinusoidal and spherical asperity contacts compared.

Rather than assuming a constant radius of curvature Hertz asperity (sometimes referred to as spherical), the asperities could be assumed to have a sinusoidal profile with the same density and peak radius of curvature. Figure 1 compares a spherical asperity contact with a sinusoidal asperity contact.

The following relations were used to convert the asperity radius and density to a frequency and amplitude of the sinusoidal asperities. The average frequency or wavelength of sinusoidal peaks can be related to the asperity density by considering two peaks occur in one square wavelength of the 3-D sinusoidal asperity [44]:

(3) $$f=\sqrt{\frac{\eta }{2}},$$

In addition, the curvature at the tip of the wavy surfaces, *R*, can then be related to the amplitude by:

(4) $$\Delta =\frac{1}{4R{\left(f\pi \right)}^{2}}.$$

The single asperity contact area and load are computed using the equations in Appendix B and substituting into Equations (1) and (2). To apply sinusoidal asperities to the statistical model, the surface separation must be known as well. Rostami and Jackson [45] derived expressions for elastic and elastic-plastic contact by extracting surface separation from a finite element model and averaging over the entire surface. The equations are for elastic contact and for elastic-plastic contact. In these equations,

(5) $$G={\left(1-\sqrt{P_{e}}\right)}^{2.5}$$

(6) $$G={\left(1-P_{ep}{}^{A_{1}P_{ep}+A_{2}}\right)}^{2.5}$$

(7) $$G=\frac{h}{\Delta },$$

(8) $$A_{1}=-0.08\ln B^{*},$$

(9) $$A_{2}=\frac{1}{15}{\left(B^{*}-1\right)}^{0.44}+0.99^{0.41\left\{B^{*}-1\right\}}-0.5,$$

(10) $$B^{*}=\frac{\Delta }{\Delta_{c}},\;$$

*G* is the nondimensional surface separation, *P_e_* and *P_ep_* are the pressure ratios relative to the required pressure for complete contact in elastic and elastic-plastic contact respectively, and Δ*_c_* is the surface amplitude above which elastic-plastic contact occurs. For a known surface separation, Equation (5) was solved numerically for the contact pressure ratio *P_ep_*, thus enabling the evaluations of Equations (1) and (2).

### 2.3. Multi-Scale Model

The third model for rough surface contact considered in this work is multi-scale and iterative [6], as it incorporates the effects of sinusoidal asperities at different scales of roughness. This is based on the Archard-stacked rough surface contact model and a later work by Ciaveralla et al. [13,23]. The surface is first transformed to the frequency domain by performing a two-dimensional FFT (Fast Fourier Transform). The number of asperities at each frequency level is calculated to determine the contact area over the entire level. For the largest scale, the area and force are defined as their nominal values. On each scale level *i*, the overall contact area and force are calculated by substituting the single asperity expressions in Appendix B into the equations

(11) $$A_{i}=\min\left(\frac{\tilde{A_{i}}}{\lambda^{2}}A_{i-1},A_{i-1}\right)$$

and

(12) $$\bar{p_{i}}=\bar{p_{i-1}}\frac{F}{A_{i-1}}.$$

In these equations, *λ* is the surface wavelength, *F* is the applied load, and *A_i_*_−1_ is the contact area on the larger scale level *i* − 1. The total contact area and pressure are the values calculated after all the scales are included.

### 2.4. Deterministic Modeling

To evaluate the models, they were compared to deterministic analyses performed by Wang [35], who used two reference surfaces in a deterministic finite element model (FEM) of rough surface contact. That study included varied resolutions on both surfaces. Both surfaces were 32 × 32 nodes with four resolutions: 1 μm, 0.5 μm, 0.25 μm, and 0.125 μm. The coarsest resolution was obtained using a profilometer, while spectral interpolation was used to create intermediate values of height between data points. A summary of the surface parameters is found in Table 1 and Table 2.

Note that although the surfaces are not perfectly Gaussian, they both could be considered approximately symmetric since the magnitude of skewness is less than ½. For the coarsest mesh the standard error of skewness (SES) is also 0.076 and the skewness for all the surfaces are within the range of 2·SES and therefore they can be considered approximately symmetric.

In order to apply any contact mechanics models, the material properties must be known. They are summarized in Table 3.

**Figure 2:**
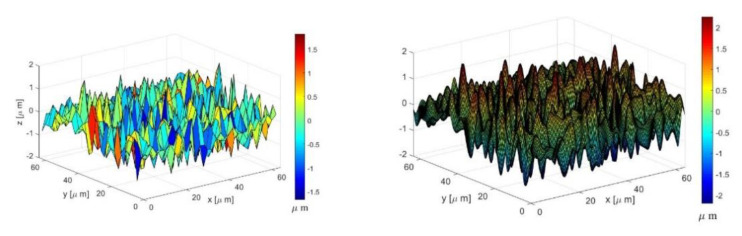
The original surface 63M and the surface after Fourier interpolation.

The surfaces came from a GAR M11 Electroforming S-22 Microfinish Comparator (Danbury, CT, USA) [43]. They were converted to the frequency domain to analyze them using the multiscale models. Figure 2 is an example of the original surface 63M and the surface after Fourier interpolation.

**Figure 3:**
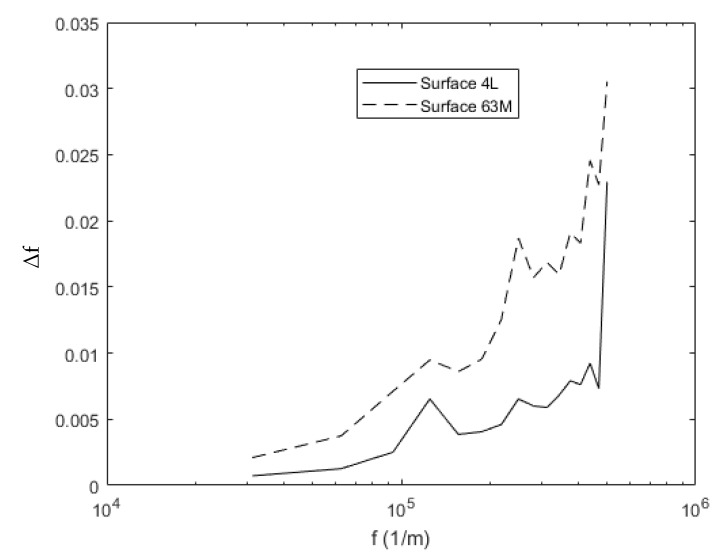
Surface spectra of surfaces 63M and 4L with 256 × 256 resolution.

Figure 3 compares surfaces 63M and 4L after the Fourier transformation. These surfaces were chosen because their roughness is not identical. It is observed that surface 63M contains much larger values of the parameter Δ*f*, which captures the aspect ratio of asperity geometry at each frequency *f* and can be calculated by multiplying the amplitude, Δ, in the frequency domain by the frequency. This quantity captures the aspect ratio of the asperity geometry at each frequency. The frequency, *f*, could be considered the scale of the asperity features; while larger values of Δ*f* indicate taller, sharper asperities. It is observed that surface 63M contains much larger values of Δ*f*.

**Figure 4:**
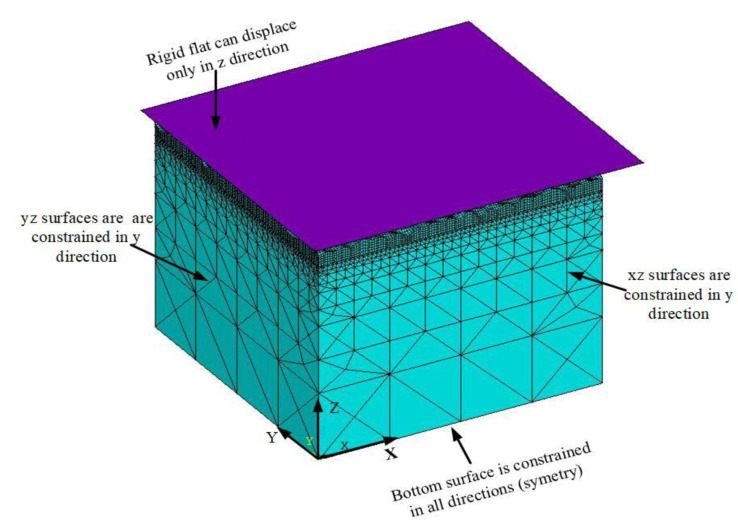
Finite element model and boundary conditions.

The three-dimensional FEM model and the boundary conditions are shown in Figure 4. All the nodes on the bottom surface are fixed in all directions. The nodes on the side surfaces are constrained in the directions perpendicular to the plane. That is, *xz* surfaces are restrained in the *y* direction, while *yz* surfaces are restrained in the x direction. The rigid flat can displace only in the z direction under normal loading. The lateral uniformly spaced mesh of contact elements on the rough surface is used to predict the real contact area. By checking the contact status of each element during post-processing, the contact area ratio is given by the number of elements in contact including both sticking and sliding divided by the total number on the rough surface. The local separation is defined as the distance between each node and the rigid flat surface, and the average gap separation is found by averaging all the values of the local separations over the entire surface. While the model includes 2% linear hardening, Kogut and Etsion [4] suggest that it has a negligible effect. Additional details of the FEM model can be found in Wang et al. [46].

## 3. Results

### 3.1. Surface 4L Analysis

**Figure 5:**
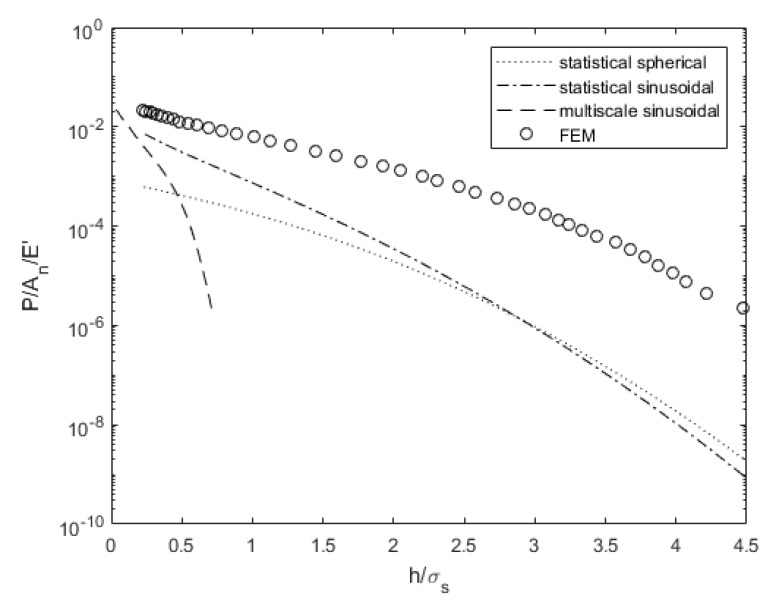
Load comparison dependent on surface separation for surface 4L.

Figure 5 compares the predicted load for a range of surface separations and contact models for surface 4L. While none of the models compare quantitively well with the deterministic results, the multiscale model predicts a much different trend than the other models. At very low surface separations, it predicts a comparable load to the statistical models. While three of the models appear to approach a common value at complete contact, the load decreases much more rapidly as the surface separation increases. Wilson et al. [47] noted the same differences and hypothesized that it accounts for the deformation of the roughness on the surface height distribution. The deterministic results predict a similar trend to the statistical models, but the load is much larger for a given surface separation. The statistical models do not consider changes in the surface distribution or the mean surface height during deformation, which could explain the discrepancies.

**Figure 6:**
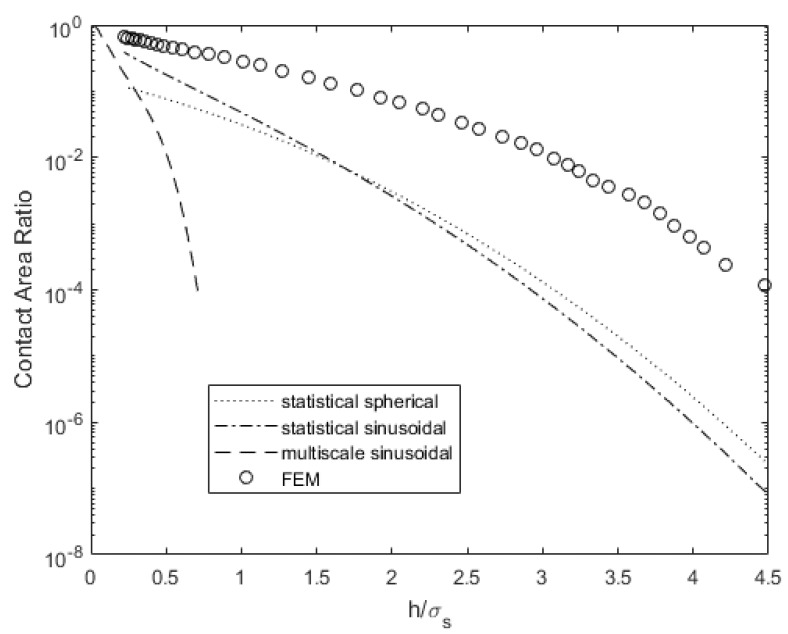
Contact area comparison for varying surface separation for surface 4L.

Figure 6 compares the contact area for a range of gaps between surface 4L and a rigid flat. The statistical and multiscale models underestimate the contact area, especially for a large gap between the surfaces. This might occur because the underlying distribution is not exactly Gaussian, which the statistical models assume. Consequently, quantities such as electrical and thermal contact resistances will be overestimated. This will result in increased power loss [48] and lower conductivity for cooling electronics [49]. When the surfaces are close, they predict a more similar contact area relative to the FEM data.

**Figure 7:**
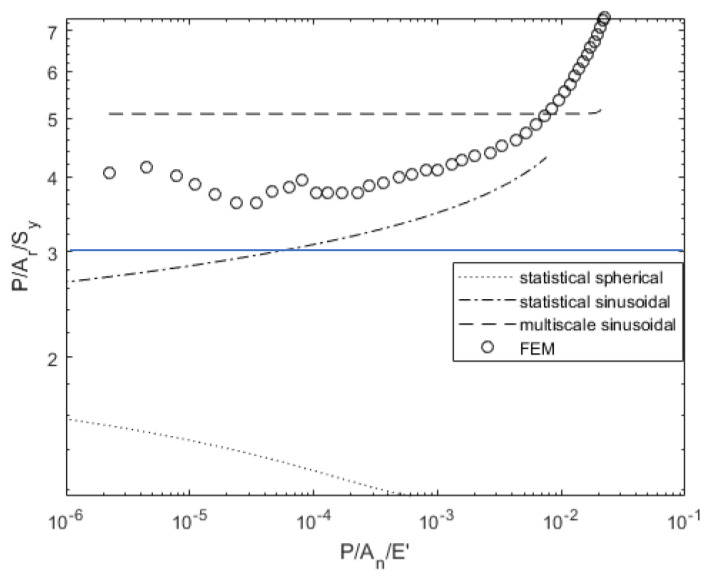
Load-dependent contact pressure variation for surface 4L.

**Figure 8:**
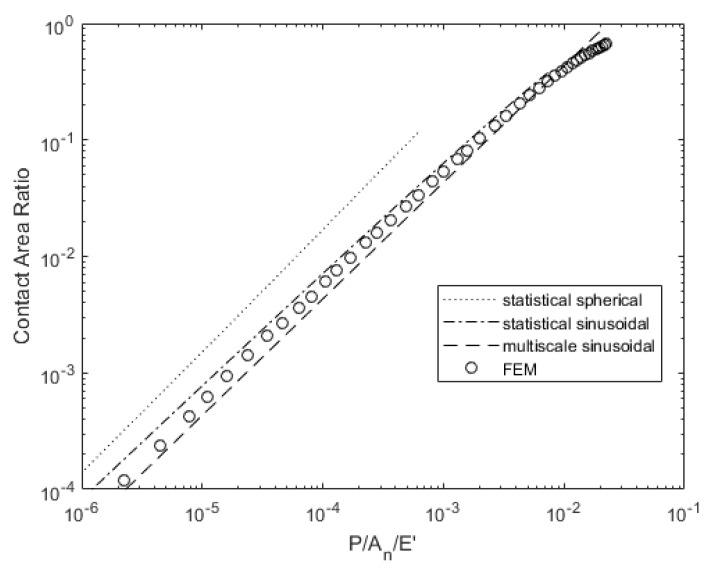
Load-dependent contact area variation for surface 4L.

Figure 7 and Figure 8 compare the contact pressure and area for a given load. The statistical models underestimate the real contact pressure *P*/*A_r_* for a given load, but the multiscale model predicts a pressure 25% greater than the deterministic results except at large loads where the model assumptions may no longer be valid. The spherical asperity model predicts a lower required load to attain a given contact area, but the models with sinusoidal asperities more closely match the deterministic solution’s required load for a given contact area.

### 3.2. Surface 63M Analysis

**Figure 9:**
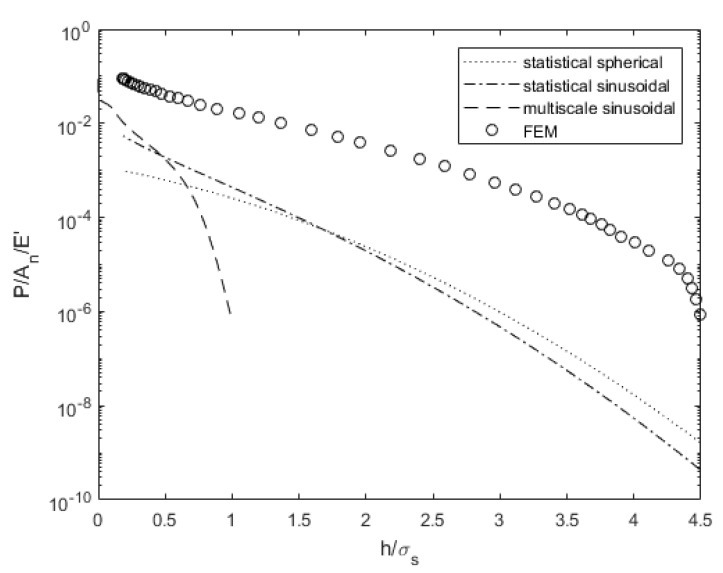
Load comparison for varying surface separation for surface 63M.

Figure 9 compares the load for varying surface separation. As with surface 4L, the statistical and multiscale models predict a lower load than the FEM results. The statistical models exhibit a similar qualitative behavior to the FEM results, but the multiscale model predicts a larger load as surface separation approaches zero. The results appear to be quite similar to those shown in Figure 4 for surface 4L (the predictions converge to a common value).

**Figure 10:**
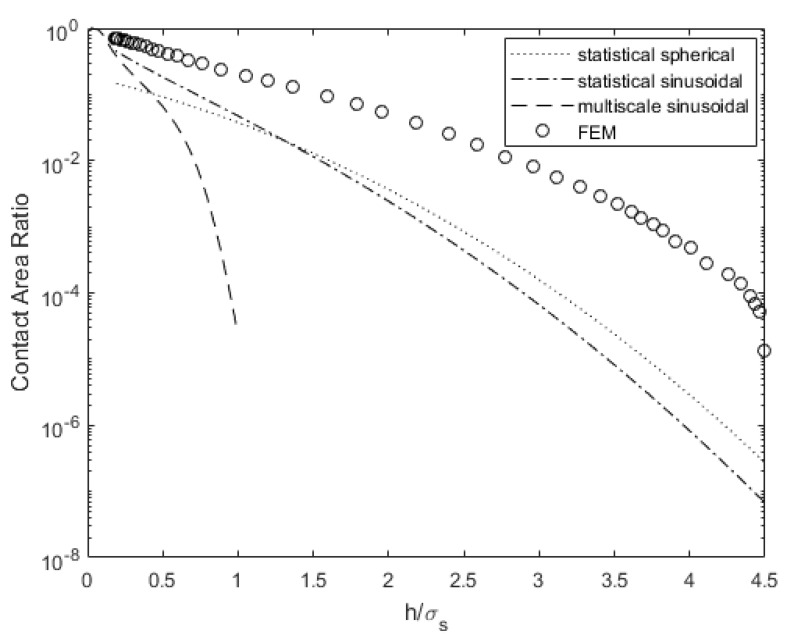
Contact area comparison for varying surface separation for surface 63M.

Figure 10 compares the contact area for a range of gaps between surface 63M and a rigid flat. The models predict a smaller contact area for a given gap relative to the FEM data but do so with different quantitative values. For very small gaps, there is not much difference in the predicted contact area. They underestimate the contact area by a greater amount for increasing *h*/*σ_s_*, but the sinusoidal asperity-based models show the best agreement overall. Larger interstitial gaps increase the predicted thermal and electrical resistances when the models are applied to real surfaces found in electronics, which results in decreased heat dissipation and electrical conduction [48,49]. Larger resistances occur due to the current and heat being bottlenecked into small isolated asperity contacts (often referred to as spreading resistance).

**Figure 11:**
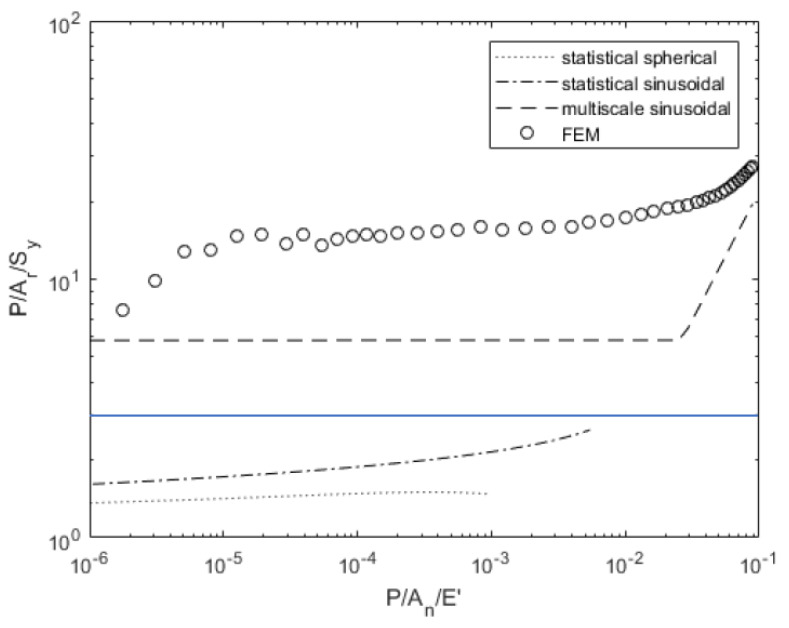
Load-dependent contact pressure variation for surface 63M.

**Figure 12:**
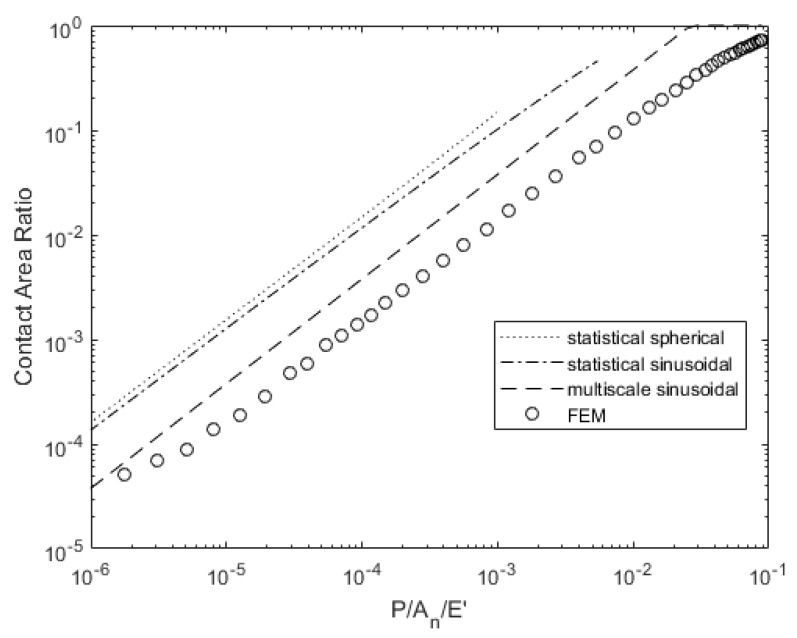
Load-dependent contact area variation for surface 63M.

Figure 11 and Figure 12 show the dependence of contact pressure and area on load. The real contact pressures predicted by the deterministic model exceed the conventional hardness value of 2.8 or 3.0*σ_y_*. Most previous texts follow the assumption that hardness (i.e., average contact pressure during fully plastic contact) is limited by 3.0*σ_y_*. However, if asperities are wavy in shape, the hardness can increase to a value well above that. This phenomena was also experimentally observed and referred to as asperity persistence [50]. It is most likely due to the shape of the contacts and the resulting stress distribution rather than hardening (increases in strength due to strain) or scale-dependent strength [51,52]. These pressures were observed in some previous works [45,53,54] and more recently by Tiwari et al. [55] to result in asperity persistence after flattening, which could explain why the statistical models predict much lower pressure and higher contact area for a given load. The multiscale model also predicts a higher contact area but does not predict any change in contact pressure until the load is large enough to result in complete contact. However, it predicts a closer match to the deterministic results than the statistical models.

## 4. Discussion

The differences in the results between the surfaces might be explained by their geometric and statistical differences and the resulting variation in plasticity that occurs in their contact. Surface 63M has approximately half the average radius of curvature, but twice the roughness. The plasticity index given by Greenwood and Williamson [1] and refined by Kogut and Etsion [4] is

(13) $$\psi =\sqrt{\frac{\sigma_{s}}{\omega_{c}}}$$

where $\sigma_{s}$ is the RMS (root mean square) roughness of the surface asperities. Increased roughness and a smaller radius of curvature usually indicate an increased susceptibility to plastic deformation as indicated by the plasticity index. This is also confirmed by the higher values of Δ*f* for surface 63M compared to 4L over all scales (see Figure 3). A higher value of Δ*f* indicates sharper asperities and a greater susceptibility to plastic deformation. This is captured by an alternative form of the plasticity index for multiscale surface roughness [33].

(14) $$\psi =\frac{{\left(f\Delta \right)}_{max}}{f\Delta_{c}}$$

The kurtosis also differed significantly between the surfaces and could influence how they deformed. Kurtosis describes how the asperity heights are distributed either closer to the mean height or away from it. A kurtosis of three corresponds to a Gaussian height distribution, while a higher value indicates that more of the asperities have heights near the mean height and a few taller asperities. These fewer taller asperities are more likely to yield because they carry higher contact pressures [56].

All models predict similar qualitative trends between applied load and contact area ratio. However, the multiscale model predicts a different relationship between surface separation and load. The statistical models predict the largest contact area ratio for a given load. The multi-scale model compares favorably with the FEM results for predicting contact pressure and contact area as a function of load but is poorly suited to predict load or contact area for a given gap between surfaces. The statistical models, while not ideal, become the best to apply when the surface separation exceeds half the RMS asperity roughness. One such application is the mixed lubrication of a cylinder liner and piston ring interface, where the surface separation is known, but the load carried by rough surface contact is not.

However, the statistical models still underestimate the load and contact area; for surfaces not considered here, the predictions may be different because the surface distribution is closer to a Gaussian distribution. When there is no nominal gap between surfaces, the multiscale model is better to use because it predicts the load at complete contact better than the statistical models, which do not adjust the asperity radius under extremely high loads or consider the interaction of asperities with adjacent asperities. This interaction between adjacent asperities can induce hydrostatic stress and resistance to plastic deformation.

The use of wavy or sinusoidal asperity models in the statistical model improves the predictions with the deterministic models relative to the spherical models. This is probably due to the sinusoidal asperity models including the coalescence effect with adjacent asperities (this is due to their assumption of periodicity). When these asperities coalesce the sustained pressure during elastic-plastic contact can be much higher than the conventional hardness of three times the yield strength. This is due to the stress becoming more hydrostatic and resistant to plastic deformation as the asperities coalesce. This is an important affect that not only occurs at high loads, but at all loads due to different scales of asperities coalescing under different loads. In addition, the small amount of hardening included in the deterministic model could account for some increase in the pressure.

## 5. Conclusions

Even though no one single model is unequivocally better than the other, one observation is clear: the models based on sinusoidal asperities better fit the deterministic results than those based on spherical asperities. Even more specifically, for predicting real contact pressure or contact area as a function of load, the multiscale model with sinusoidal asperities should be used. While for predicting contact force as a function of surface separation, the statistical models with sinusoidal asperity models appear to be the most effective.

Overall, the comparisons with the deterministic models appear to be inconsistent, especially for load as a function of surface separation. It may be possible to improve these comparisons by including more details, such as non-Gaussian asperity distributions and distributions that consider variation of the radius of curvature. In addition, the small amount of hardening included in the deterministic model but not in the statistical and multiscale models could account for some of these discrepancies. This is considered beyond the aim of the current work but can be investigated in the future.

## Figures and Tables

**Table 1:** Statistical parameters for surface 4L for difference node densities.

	32 × 32	64 × 64	128 × 128	256 × 256
*R* (µm)	6.9	1.335	0.989	0.958
*η* (1/µm^2^)	0.0234	0.1074	0.1523	0.168
*σ_s_* (μm)	0.1615	0.223	0.246	0.261
Skewness	0.1	0.086	0.086	0.0864
Kurtosis	2.38	2.59	2.54	2.53

**Table 2:** Statistical parameters of surface 63M.

	32 × 32	64 × 64	128 × 128	256 × 256
*R* (µm)	2.75	0.8	0.567	0.518
*η* (1/µm^2^)	0.0327	0.11	0.1494	0.16
*σ_s_* (μm)	0.413	0.476	0.495	0.501
Skewness	0.137	0.114	0.1134	0.113
Kurtosis	3.32	3.46	3.423	3.42

**Table 3:** Material properties for the reference surfaces.

Property	Value
*E*	200 GPa
*ν*	0.3
*S_y_*	1 GPa

## Data Availability

Data sharing is not applicable to this article.

## Nomenclature

*A_n_*	Nominal contact area
*A_r_*	Real contact area
$\bar{A}$	Single asperity contact area
*ω*	Interference between surfaces
*ω_c_*	Critical interference
*S_y_*	Yield strength
*E*	Elastic modulus
*ν*	Poisson’s ratio
*E’*	Effective elastic modulus, *E*/(1 − *ν*^2^)
$\bar{F}$	Single asperity contact force
*η*	Asperity density
*R*	Asperity radius
*σ_s_*	RMS Asperity Height
*f*	Spatial frequency
λ	Wavelength of sinusoidal surface (1/*f*)
Δ	Amplitude of sinusoidal surface
$\bar{p}$	Average pressure over surface
*p**	Average pressure for complete elastic contact
*p*_ep_*	Average pressure for complete elastic-plastic contact

## Appendix A. Summary of the JG Model

The single asperity contact area and load using the JG model are given by:

(A1) $$\bar{A}=\pi R\omega {\left(\frac{\omega }{1.9\omega_{c}}\right)}^{B}$$

(A2) $$\bar{P}=\bar{P_{c}}\left\{\left[\exp(-0.25{\left(\frac{\omega }{\omega_{c}}\right)}^{\frac{5}{12}})\right]{\left(\frac{\omega }{\omega_{c}}\right)}^{\frac{3}{2}}+\frac{4H_{G}}{CS_{y}}\left[1-\exp(-0.04{\left(\frac{\omega }{\omega_{c}}\right)}^{\frac{5}{9}})\right]\frac{\omega }{\omega_{c}}\right\},$$

where,

(A3) $$B=0.14e^{23e_{y}},$$

(A4) $$e_{y}=\frac{S_{y}}{E\prime },$$

(A5) $$\frac{H_{G}}{S_{y}}=2.84-0.92\left[1-\cos(\frac{\pi a}{R})\right],$$

(A6) $$\bar{P_{c}}=\frac{4}{3}{\left(\frac{R}{E\prime }\right)}^{2}{\left(\frac{C\pi S_{y}}{2}\right)}^{3}$$

(A7) $$\omega_{c}=R{\left(\frac{\pi CS_{y}}{2E\prime }\right)}^{2},$$

where $C=1.295e^{0.736\nu }.$ In these equations, *R* is the asperity radius, *ω* is the asperity interference, *ω_c_* is the interference above which elastic-plastic contact occurs, *S_y_* is the yield strength, *E*′ is the equivalent elastic modulus, and *ν* is Poisson’s ratio.

## Appendix B. Contact Area and Load for the Sinusoidal Asperity Model

Johnson, Greenwood, and Higginson (JGH) [12] developed a piecewise defined function between pressure and contact area for a single asperity under elastic loading. In their equations, $\bar{p}$ is the average contact pressure, and *p^*^* is the pressure required for complete contact, which is given by

(A8) $$p^{*}=\sqrt{2}\pi E\prime f\Delta .$$

The ratio of the contact pressure and the pressure required for complete contact is called *P_e_*. JGH were not able to derive a closed form solution, but rather provided two asymptotic solutions based on Hertz contact. For *P_e_* << 1,

(A9) $${\left({\bar{A}}_{JGH}\right)}_{1}=\pi \lambda^{2}{\left[\frac{3P_{e}}{8\pi }\right]}^{\frac{2}{3}},$$

while for large values of *P_e_*,

(A10) $${\left({\bar{A}}_{JGH}\right)}_{2}=\frac{\lambda^{2}}{2}\left(1-\frac{3}{2\pi }\left[1-P_{e}\right]\right).$$

Jackson and Streator [6] fitted a polynomial that combined the two equations above, using the experimental data from Johnson et al:

For *P_e_* < 0.8,

(A11) $$\tilde{A}={\left({\bar{A}}_{JGH}\right)}_{1}\left(1-P_{e}{}^{1.51}\right)+{\left({\bar{A}}_{JGH}\right)}_{2}P_{e}{}^{1.04}$$

For *P_e_* ≥ 0.8,

(A12) $$\tilde{A}={\left({\bar{A}}_{JGH}\right)}_{2}$$

These equations neglect asperity yielding, so an elastic-plastic model for large loads developed by Krithivasan and Jackson [24] is employed. The equation for the contact area at low pressures when plastic deformation occurs is

(A13) $$A_{p}=2{\left(\frac{A_{c}}{2}\right)}^{\frac{1}{1+d}}{\left(\frac{3\bar{p}}{4Cf^{2}S_{y}}\right)}^{\frac{d}{1+d}},$$

where,

(A14) $$d=3.8{\left(\frac{E\prime f\Delta }{S_{y}}\right)}^{0.11},$$

and

(A15) $$A_{c}=\frac{2}{\pi }{\left(\frac{CS_{y}}{8E\prime f^{2}\Delta }\right)}^{2}.$$

is the critical area at which elastic-plastic contact begins. Complete contact occurs at much lower pressures compared to a purely elastic model; that value is *p*_ep_*_._ The ratio of the contact pressure, $\bar{p}$, and *p*_ep_* is *P_ep_*. The equation linking contact area and load over low pressures and high pressures is:

(A16) $$\bar{A}=A_{p}\left(1-P_{ep}{}^{1.51}\right)+{\left({\bar{A}}_{JGH}\right)}_{2}P_{ep}{}^{1.04}.$$

In this equation, the value of ${\left({\bar{A}}_{JGH}\right)}_{2}$ is calculated by replacing *P_e_* with *P_ep_*. They also derived a critical average contact pressure below which contact remains in the elastic regime. Because that was derived from spherical contact, Jackson et al. [53] derived a new model by computing the critical interference Δ*_c_*, above which elastic-plastic relations are used. Their corrected expression for it as published by Ghaednia et al. [47] is

(A17) $$\Delta_{c}=\frac{\sqrt{2}S_{y}}{E\prime f\pi \left[3e^{-\frac{2}{3}\left(\nu +1\right)}+2\left(\frac{1-2\nu }{1-\nu }\right)\right]}.$$

Using this value of critical interference, a new equation for *p*_ep_* that relates it to *p** was fitted to the FEM data from [24],

(A18) $$\frac{P^{*}{}_{ep}}{p^{*}}=0.992^{\left[{\left\{\frac{\Delta }{\Delta_{c}}\right\}}^{\frac{10}{3}{\left(\frac{\Delta }{\Delta_{c}}\right)}^{-0.39}+\frac{9}{4}\nu^{4}+0.64}-1\right]}$$
